# The OmpL37 Surface-Exposed Protein Is Expressed by Pathogenic *Leptospira* during Infection and Binds Skin and Vascular Elastin

**DOI:** 10.1371/journal.pntd.0000815

**Published:** 2010-09-07

**Authors:** Marija Pinne, Henry A. Choy, David A. Haake

**Affiliations:** 1 Research Service, 151, Veterans Affairs Greater Los Angeles Healthcare System, Los Angeles, California, United States of America; 2 Department of Medicine, David Geffen School of Medicine, University of California Los Angeles, Los Angeles, California, United States of America; 3 Division of Infectious Diseases, 111F, Veterans Affairs Greater Los Angeles Healthcare System, Los Angeles, California, United States of America; 4 Department of Urology, David Geffen School of Medicine, University of California Los Angeles, Los Angeles, California, United States of America; 5 Department of Microbiology, Immunology and Molecular Genetics, University of California Los Angeles, Los Angeles, California, United States of America; Institut Pasteur, France

## Abstract

Pathogenic *Leptospira* spp. shed in the urine of reservoir hosts into freshwater can be transmitted to a susceptible host through skin abrasions or mucous membranes causing leptospirosis. The infection process involves the ability of leptospires to adhere to cell surface and extracellular matrix components, a crucial step for dissemination and colonization of host tissues. Therefore, the elucidation of novel mediators of host-pathogen interaction is important in the discovery of virulence factors involved in the pathogenesis of leptospirosis. In this study, we assess the functional roles of transmembrane outer membrane proteins OmpL36 (LIC13166), OmpL37 (LIC12263), and OmpL47 (LIC13050), which we recently identified on the leptospiral surface. We determine the capacity of these proteins to bind to host tissue components by enzyme-linked immunosorbent assay. OmpL37 binds elastin preferentially, exhibiting dose-dependent, saturating binding to human skin (K_d_, 104±19 nM) and aortic elastin (K_d_, 152±27 nM). It also binds fibrinogen (K_d_, 244±15 nM), fibrinogen fragment D (K_d_, 132±30 nM), plasma fibronectin (K_d_, 359±68 nM), and murine laminin (K_d_, 410±81 nM). The binding to human skin elastin by both recombinant OmpL37 and live *Leptospira interrogans* is specifically enhanced by rabbit antiserum for OmpL37, suggesting the involvement of OmpL37 in leptospiral binding to elastin and also the possibility that host-generated antibodies may promote rather than inhibit the adherence of leptospires to elastin-rich tissues. Further, we demonstrate that OmpL37 is recognized by acute and convalescent leptospirosis patient sera and also by *Leptospira*-infected hamster sera. Finally, OmpL37 protein is detected in pathogenic *Leptospira* serovars and not in saprophytic *Leptospira*. Thus, OmpL37 is a novel elastin-binding protein of pathogenic *Leptospira* that may be promoting attachment of *Leptospira* to host tissues.

## Author Summary

Leptospirosis is a potentially fatal disease in humans and livestock caused by *Leptospira* bacteria. Effective antibiotic treatment depends on timely, accurate diagnosis. However, current diagnostic and vaccine options are limited by their specificity for the lipid-sugar coat of leptospires, which varies among 200 serum-reactive groups. We aim to understand how leptospires infect a host, and in turn, to develop broadly effective diagnostic and immunization products. We recently described OmpL37, a new protein on the surface of leptospires. Here, we show it is made by pathogenic strains, suggesting it can be a target for detecting and protecting against a wide range of *Leptospira*. Moreover, leptospirosis patients and hamsters infected with leptospires make antibodies against OmpL37. Purified OmpL37 binds host proteins, including human elastin, fibrinogen, fibronectin, and mouse laminin. Although other leptospiral proteins bind multiple host proteins, OmpL37 has novel preferential affinity for skin and aorta elastin, suggesting a role in a common route of transmission through abraded skin and exposed blood vessels. Indeed, OmpL37 binding and leptospiral attachment to elastin are both enhanced by OmpL37 antiserum, further implicating a possible role for OmpL37 during infection. Thus, OmpL37 may mediate host attachment and has potential clinical application with a broad range of *Leptospira*.

## Introduction

Leptospirosis is a zoonosis caused by pathogenic *Leptospira* spp. transmitted from reservoir hosts (typically rodents) to humans via water contaminated by infected animals and has a significant impact on public health throughout the developing world [1]–[4]. Leptospirosis also has significant adverse effects on the agricultural industry by causing abortions, infertility, and death in livestock [5], [6]. After being shed in the urine of a reservoir host animal, leptospires can persist in freshwater or soil until contact with abraded skin or mucous membranes of a new host occurs. The resulting infection is potentially fatal, and is frequently characterized by jaundice, renal failure, and/or pulmonary hemorrhage [1], [4], [7]. Large outbreaks of leptospirosis occur in tropical and subtropical regions after heavy rainfall and the dispersal of leptospires in contaminated water [3], [8]. Current vaccines against leptospirosis target the lipopolysaccharide (LPS) coat of the leptospires, which is highly variable; this variation is thought to be the major antigenic determinant defining the differences between approximately 230 serovars that contribute to serovar-specific immunity [6], [9]. In contrast, vaccines directed towards well-conserved leptospiral outer membrane proteins (OMPs) [10], [11] would have an advantage in inducing cross-protective immunity [12].

The leptospiral lifecycle involves interactions with host tissues at multiple stages of infection, including: (i) entering the host, (ii) evading its immune response, and (iii) adhering to tissues [13]–[15]. Identification and characterization of novel proteins that mediate the stage-specific interactions with the host are essential for the understanding of leptospiral pathogenesis, and in the development of diagnostic and protective antigens for leptospirosis. Pathogenic leptospires have been shown to bind to a variety of host ligands, including fibronectin, fibrinogen, collagen, laminin, elastin, and proteoglycans, indicating that cell surface and extracellular matrix (ECM)-binding OMPs, or adhesins, are likely to be expressed by the spirochetes [16]–[21]. It is possible that leptospires express distinct adhesins at different stages of infection, including initial attachment, dissemination, and colonization. Many leptospiral proteins, including LigA/B, Lsa21, Lsa27, Lsa63, Lsa24 (LfhA/LenA), LenB to F, LipL32, Lp95, TlyC, and LipL53, have been shown to have affinity for host ligands *in vitro* [18], [19], [21]–[32]. However, it is unclear to what extent these putative adhesins mediate interactions of leptospires with cell surface and ECM proteins. Only Lsa24, LigA/B, and Lsa63 have been tested for their capacity to inhibit leptospiral adherence to ECM proteins [18], [21], [24], [32]. In each case, only partial inhibition was observed, suggesting that additional fibronectin-, laminin-, collagen-, and elastin-binding proteins likely exist in *Leptospira* [18], [21], [24], [32].

In this study, we investigated whether the surface-exposed proteins in *Leptospira*, OmpL36 (LIC13166), OmpL37 (LIC12263), and OmpL47 (LIC13050) that we recently described [33] can bind to any host ligands. We now report that OmpL37 is the first leptospiral protein found to have pronounced specificity for human skin elastin. OmpL37 exhibits strong, saturating binding to skin elastin with one of the highest affinities of all leptospiral ligand-binding proteins described. In addition, OmpL37 binds efficiently to human aortic elastin, fibrinogen, fibrinogen fragment D, and to a lesser extent laminin and plasma fibronectin. OmpL47 also binds to laminin, plasma fibronectin, fibrinogen, fibrinogen fragment D, along with collagen type III, and aortic elastin, but showing much lower activities than OmpL37. OmpL36 shows no binding to any of the host tissue components investigated.

Elastin is a connective tissue component of ECM responsible for the elasticity and resilience of skin, lung, blood vessels, uterus, placenta, and other tissues [34]–[36]. These elastin-rich tissues are highly relevant to leptospirosis as infection includes entry through skin abrasions or mucous membranes, dissemination through the circulation, and attachment to vascular, renal, pulmonary, uterine, and other tissues. Until our discovery of the elastin-binding properties of OmpL37, only LigB was known to have the capacity to bind elastin and tropoelastin [21]. We also show that OmpL37 antiserum can enhance the binding to skin elastin by both live *Leptospira* and recombinant OmpL37, suggesting that leptospiral binding to elastin is at least partially mediated by OmpL37. Expression of OmpL37 during infection is confirmed with the recognition of OmpL37 by sera from *Leptospira*-infected hamsters and also acute and convalescent leptospirosis patients. While the gene for an OmpL37 homologue is present in saprophytic leptospires, OmpL37 is detectable only in pathogenic *Leptospira* serovars. Taken together, our data suggest that OmpL37 is an elastin-binding protein of *Leptospira* with potential roles in leptospirosis, including the attachment to elastin-rich tissues, such as the dermis, vasculature, and lungs.

## Materials and Methods

### Ethics statement

This study was conducted according to the principles expressed in the Declaration of Helsinki. The study was approved by the Institutional Review Board of the Research and Development Committee, VA Greater Los Angeles Healthcare System, Research Service (PCC # 2008-121778).

All animals were handled in strict accordance with good animal practice as defined by the relevant national and/or local animal welfare bodies, and all animal work was approved by the VA Greater Los Angeles Healthcare System, Research Service (PCC # 2009-010088).

### Bacterial strains and growth conditions

*Leptospira interrogans* serovar Copenhageni strain Fiocruz L1-130 was isolated from a patient during a leptospirosis outbreak in Salvador, Brazil [8]. *L. interrogans* serovar Pomona strain PO-01, *L. kirscheneri* serovars Mazdok strain 5621 and Grippotyphosa strain RM52, *L. borgpetersenii* serovars Tarrasovi strain Perepelicin and Javanica strain Veldrat Bataviae 46, *Leptonema illini* strain 3055, *Leptospira weilii* serovar Celledoni strain Celledoni, *L. wolbachii* serovar Biflexa strain codice, *L. inadai* serovar Lyme strain 10, and *L. biflexa* serovar Patoc strain Patoc 1 were obtained from the National Leptospirosis Reference Center (National Animal Disease Center, Agricultural Research Service, U.S. Department of Agriculture, Ames, Iowa). Leptospires were cultivated in Ellinghausen-McCullough-Johnson-Harris (EMJH) medium supplemented with 1% rabbit serum (Rockland Immunochemicals, Gilbertsville, PA) and 100 µg/ml 5-fluorouracil at 30°C [37].

### Antibodies and serum samples

The polyclonal rabbit sera specific for OmpL37, OmpL47, and OmpL54 have been described previously [33]. Immunoglobulins G (IgG) from OmpL37 and OmpL47 antisera were purified by Melon Gel IgG spin purification kit (Thermo Scientific, Rockford, IL) according to manufacturer's instructions. LipL32 monoclonal antibody 1D9 [38], [39] was a kind gift from Dr. José Antonio Guimarães Aleixo (Universidade Federal De Pelotas, Pelotas, Brazil). Pooled sera from infected Syrian hamsters (Harlan Laboratories) were obtained ten days following intradermal challenge of one month-old animals with *L. interrogans* L1-130. As a negative control, a serum sample from a hamster injected intradermally with EMJH was collected after 12 days. Patient sera from leptospirosis outbreaks in 1996 and 1997 in Salvador, Brazil, were kindly provided by Dr. Albert I. Ko (Oswaldo Cruz Foundation, Salvador, Bahia, Brazil). Acute and convalescent samples from the same patients were prepared by pooling sera from 13 individuals with laboratory-confirmed leptospirosis. Normal human serum was obtained from Thermo Scientific and Millipore (Billerica, MA).

### Cloning, expression, and purification of recombinant LIC10091

The gene encoding potential outer membrane lipoprotein, LIC10091 (LipL40) [40], was amplified from Fiocruz L1-130 DNA using forward primer, 5′- TTCGCATATGAAAACGCCTCCTCCTAAAG -3′, and reverse primer, 5′- TAAAATCTCGAGTTTCAAAACTTCTACGGGC- 3′, by PCR conditions as described for *ompL36, ompL37,* and *ompL47* [33]. PCR product was digested with *Nde*I and *Xho*I (New England BioLabs, Ipswich, MA), cloned into *Nde*I- and *Xho*I- digested expression vector, pET-20b(+) (Novagen, San Diego, CA), and purified as previously described for OmpL36, OmpL37, and OmpL47 [33].

### Gel electrophoresis and immunoblotting

Protein samples were boiled for 5 min in Novex NuPAGE sample buffer (Invitrogen, Carlsbad, CA) in the presence of 2.5% β-mercapthoethanol and separated in Bis-Tris 4–12% polyacrylamide gradient NuPAGE gels (Invitrogen).

For immunoblotting, proteins were transferred to a polyvinylidene difluoride (PVDF) Immobilon-P membrane (Millipore) and probed with rabbit polyclonal antisera, Syrian hamster sera, or leptospirosis patient sera. Bound antibodies were detected using horseradish peroxidase (HRP)-conjugated anti-rabbit IgG (GE Lifesciences, Buckinghamshire, England), anti-Syrian hamster IgG (Jackson Immuno Research, West Groove, PA), or anti-human IgG (Sigma-Aldrich, St. Louis, MO), respectively. Immunoblots were visualized by enhanced chemiluminescence reagents according to the manufacturer's instructions (Thermo Scientific).

### Enzyme-linked immunosorbent assay (ELISA) of binding to host ligands by OmpL proteins

Host ligands included human plasma fibronectin (Sigma-Aldrich), human plasma fibronectin 30-kDa proteolytic fragment (heparin-binding domain, Sigma-Aldrich), human plasma fibronectin 45-kDa proteolytic fragment (gelatin-binding domain, Sigma-Aldrich), human fibroblast fibronectin (Calbiochem, La Jolla, CA), human plasma fibrinogen (HYPHEN BioMed, France), human plasma fibrinogen fragment D (HYPHEN BioMed), murine laminin (Sigma-Aldrich), bovine skin collagen type I (Sigma-Aldrich), human placenta collagen type III (Sigma-Aldrich), human placenta collagen type IV (Sigma-Aldrich), soluble human skin elastin (Elastin Products Company, Owensville, MO), soluble human aorta elastin (Sigma-Aldrich), bovine kidney heparan sulfate (Sigma-Aldrich), shark cartilage chondroitin sulfate (Sigma-Aldrich), fetal calf serum fetuin (Sigma-Aldrich), and bovine serum albumin (BSA, Sigma-Aldrich). Ultra-high binding Immulon 4HBX microtiter plates (Thermo Scientific) were coated with 1 µg of host ligand in 0.1 ml of phosphate buffered saline (PBS), pH 7.2, and incubated overnight at 4°C. Fresh (stored for less than 4 months at 4°C) recombinant OmpL36, OmpL37, OmpL47 [33], and LIC10091 (used as a negative control) binding to individual ligands was assessed by ELISA. Briefly, non-specific binding sites were blocked with Protein-Free Blocking buffer (PFBb; Thermo Scientific) for 1 h at room temperature and 1 µg of recombinant protein in 0.1 ml of PFBb was added per well and incubated for 1 h at 37°C. For assays of ligand binding as a function of leptospiral protein concentration, serial dilutions of recombinant OmpL37 and OmpL36 (negative control) ranging from 0 to 2 µM in 0.1 ml of PFBb were added to wells and incubated for 1 h at 37°C. Wells were washed three times with PBS, pH 7.2, and bound protein was detected by probing with anti-His Tag monoclonal antibody (5 Prime, Gaithersburg, MD), developing with HRP-conjugated anti-mouse IgG (Novagen) and a tetramethyl benzidine substrate (Thermo Scientific), and recording by spectrophotometry at 450 nm. For saturating binding the apparent dissociation constant (K_d_) was estimated as the concentration of OmpL37 resulting in half-maximal binding.

For assays assessing effects of antibodies on binding of recombinant OmpL37 to skin elastin, recombinant OmpL37 was pre-incubated either with serum against OmpL37 or OmpL47 (negative control) at 1∶2500 dilution or purified IgG at 1∶40 to 1∶640 serial dilutions in PFBb at room temperature for 30 min. In addition, OmpL37 was pre-incubated with convalescent leptospirosis patient sera or *Leptospira*-infected hamster sera at 1∶640 to 1∶10,240 serial dilutions in PFBb at room temperature for 1 h. Then mixtures containing 0.5 µg of OmpL37 in 0.1 ml of PFBb were added to microtiter wells, incubated for 1 h at 37°C, and bound protein detected as described above.

### ELISA of immobilized OmpL37 binding to freely soluble host ligands

Oneµg of recombinant OmpL37 or OmpL36 (negative control) in 0.1 ml PBS, pH 7.2, was used to coat Immulon 4HBX microtiter wells overnight at 4°C. Non-specific binding sites were blocked with PFBb and 1 µg of human plasma fibronectin, human plasma fibrinogen, human plasma fibrinogen fragment D, murine laminin, bovine skin collagen type I, human collagen type IV, or human skin elastin in 0.1 ml of PFBb was added and incubated for 1 h at room temperature. After three washes with PBS, pH 7.2, OmpL37-bound host ligands were detected by probing with anti-human fibronectin rabbit antibody (Sigma-Aldrich), anti-human fibrinogen rabbit IgG (HYPHEN BioMed), anti-laminin rabbit IgG (Sigma-Aldrich), anti-collagen type I monoclonal antibody (Clone COL-1, Sigma-Aldrich), anti-collagen type IV monoclonal antibody (Clone COL-94, Sigma-Aldrich), or anti-elastin rabbit IgG (Santa Cruz Biotechnology, Inc., Santa Cruz, CA), then adding HRP-conjugated anti-mouse IgG (Novagen) or HRP-conjugated anti-rabbit IgG (GE Lifesciences) and developing as described above.

### ELISA of leptospiral binding to skin elastin

Microtiter plates were coated with human skin elastin and non-specific binding sites were blocked as described above. *L. interrogans* cultures were harvested by centrifugation at 2000× *g* for 15 min at room temperature and resuspended in PBS-5 mM MgCl_2_ to a final concentration of 1×10^9^ cells/ml. To assess the effect of the OmpL37 antibodies on leptospiral binding to elastin, serial dilutions (1∶40 to 1∶1280) of anti-OmpL37 or anti-OmpL54 (used as a negative control) were mixed with the leptospires or PBS-5 mM MgCl_2_ and 1×10^8^ cells in 0.1 ml of PBS-5 mM MgCl_2_ were added to the microtiter wells. For experiments assessing the inhibition of leptospiral binding by recombinant OmpL37, prior to the addition of leptospires, 0.5 µM of recombinant protein in 0.1 ml of PFBb was either added directly to elastin-coated microtiter wells or pre-incubated with anti-OmpL37 or anti-OmpL47 (negative control) at a 1∶500 dilution for 30 min at room temperature and added to the microtiter wells. Plates were incubated for 1 h at 37°C, and washed three times with PBS. After addition of leptospires, plates were incubated at 30°C for 90 min, unbound leptospires were removed by four washes with PBS-5 mM MgCl_2_, and adherent cells were fixed with methanol at −20°C for 10 min. Elastin-bound leptospires were detected by probing with LipL32 monoclonal antibody 1D9 and developing as described above.

To measure the effect of OmpL37 antibodies on the binding of immobilized leptospires to freely soluble skin elastin, 1×10^8^ of *L. interrogans* in PBS-5 mM MgCl_2_ were allowed to adhere to Immulon 4HBX microtiter wells for 90 min at 30°C, washed twice with PBS-5 mM MgCl_2_, and non-specific binding sites were blocked with PFBb for 30 min at room temperature. Rabbit sera for OmpL37 and OmpL47 (negative control) diluted 1∶40 and 1∶2560 in PBS-5 mM MgCl_2_ were added to the wells and incubated for 90 min at room temperature, followed by three washes with PBS-5 mM MgCl_2_. One µg of human skin elastin in 0.1 ml PBS-5 mM MgCl_2_ was added and incubated overnight at 4°C. Wells were washed three times with PBS, pH 7.2, and cell-bound complexes were fixed with methanol at −20°C for 10 min, followed by additional blocking with PFBb. *Leptospira*-bound elastin was detected by probing with anti-elastin mouse serum (Novus Biologicals, Littleton, CO) and developing as described above.

## Results

### Recombinant OmpL37 binds host tissue components

**Figure 1:**
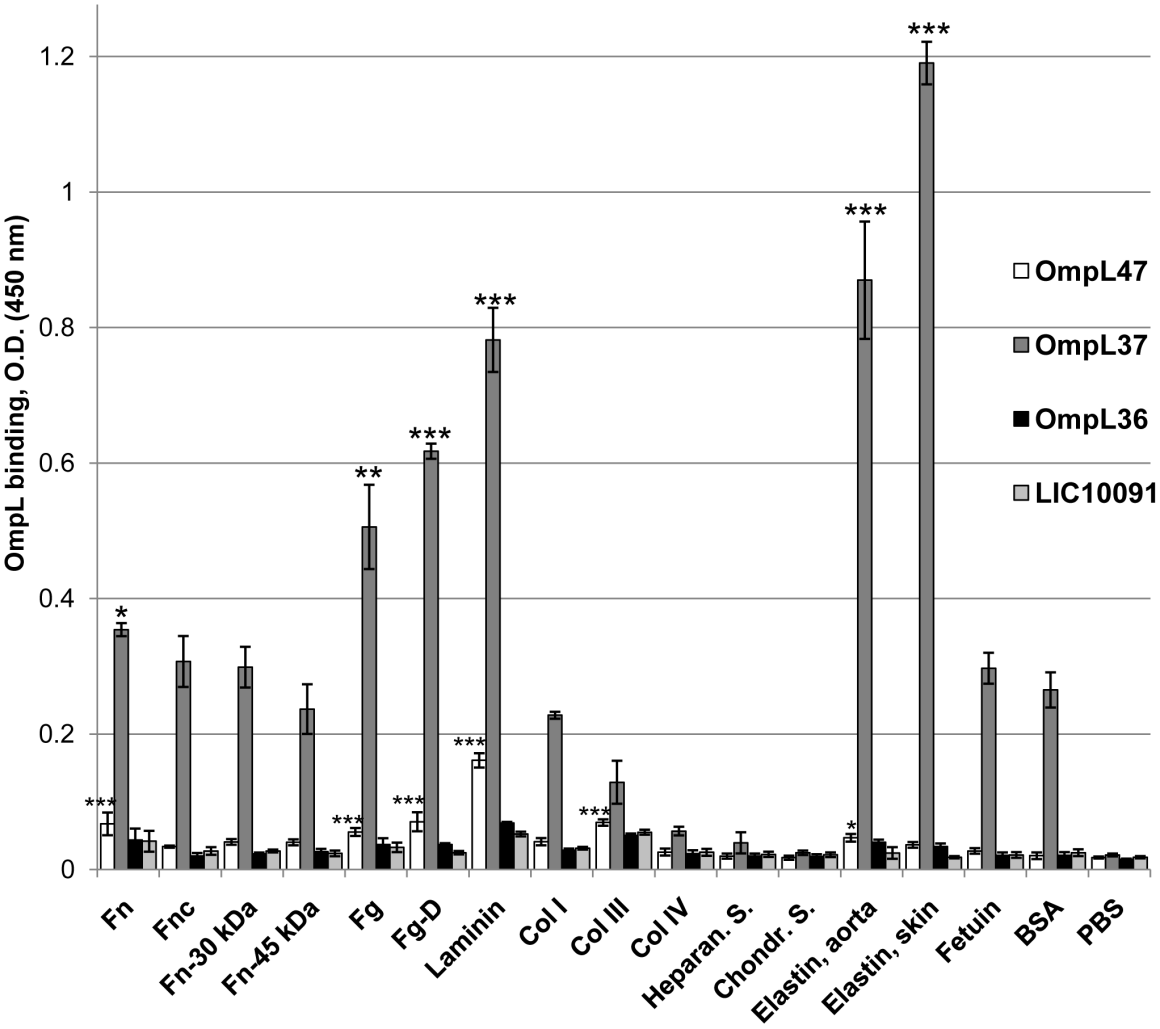
Binding of recombinant Omp36, OmpL37, and OmpL47 to host tissue components. Microtiter wells were coated with 1 µg of plasma fibronectin (Fn), fibroblast cellular fibronectin (Fnc), heparin-binding domain of plasma fibronectin (Fn-30 kDa), gelatin-binding domain of plasma fibronectin (Fn-45 kDa), plasma fibrinogen (Fg), plasma fibrinogen fragment D (Fg-D), laminin (Lm), collagen type I (Col I), collagen type III (Col III), collagen type IV (Col IV), kidney heparan sulfate (Heparan. S.), cartilage chondroitin sulfate (Chondr. S.), aorta elastin, skin elastin, fetuin, and BSA. One microgram of recombinant protein was added per well and binding was measured by ELISA. Data represent the mean absorbance at 450 nm ± the standard deviation of three independent experiments. The binding of recombinant proteins to tissue components was compared to their binding to BSA by Student's two-tailed *t* test (*** P<0.001, ** P<0.01, * P<0.05).

To investigate the capacities of our recently described surface-exposed transmembrane OmpL proteins [33] to interact with host tissue ligands, soluble recombinant OmpL36, OmpL37, and OmpL47 were assessed for binding to immobilized host components. BSA and the highly glycosylated serum protein, fetuin, were used as controls for non-specific binding. Soluble recombinant LIC10091 was used as a non-binding protein control. OmpL37 exhibited significant binding to ECM components, such as human skin (P<0.001 compared to BSA) and aorta elastin (P<0.001), laminin (P<0.001), fibrinogen (P<0.01), fibrinogen fragment D (P<0.001), and plasma fibronectin (P<0.05) (Fig. 1). OmpL37 binding to the 30-kDa and 45-kDa fragments of plasma fibronectin, fibroblast fibronectin, collagen types I, III, and IV, heparan sulfate, and chondroitin sulfate was not statistically significant (Fig. 1). OmpL47 also showed significant binding to laminin (P<0.001), fibrinogen (P<0.001), fibrinogen fragment D (P<0.001), plasma fibronectin (P<0.001), collagen III (P<0.001), and aorta elastin (P<0.05). However, the OmpL47 activities were much lower than those observed for OmpL37 and were not investigated further. None of the other recombinant proteins exhibited significant binding to any of the host tissue components investigated (Fig. 1).

**Figure 2:**
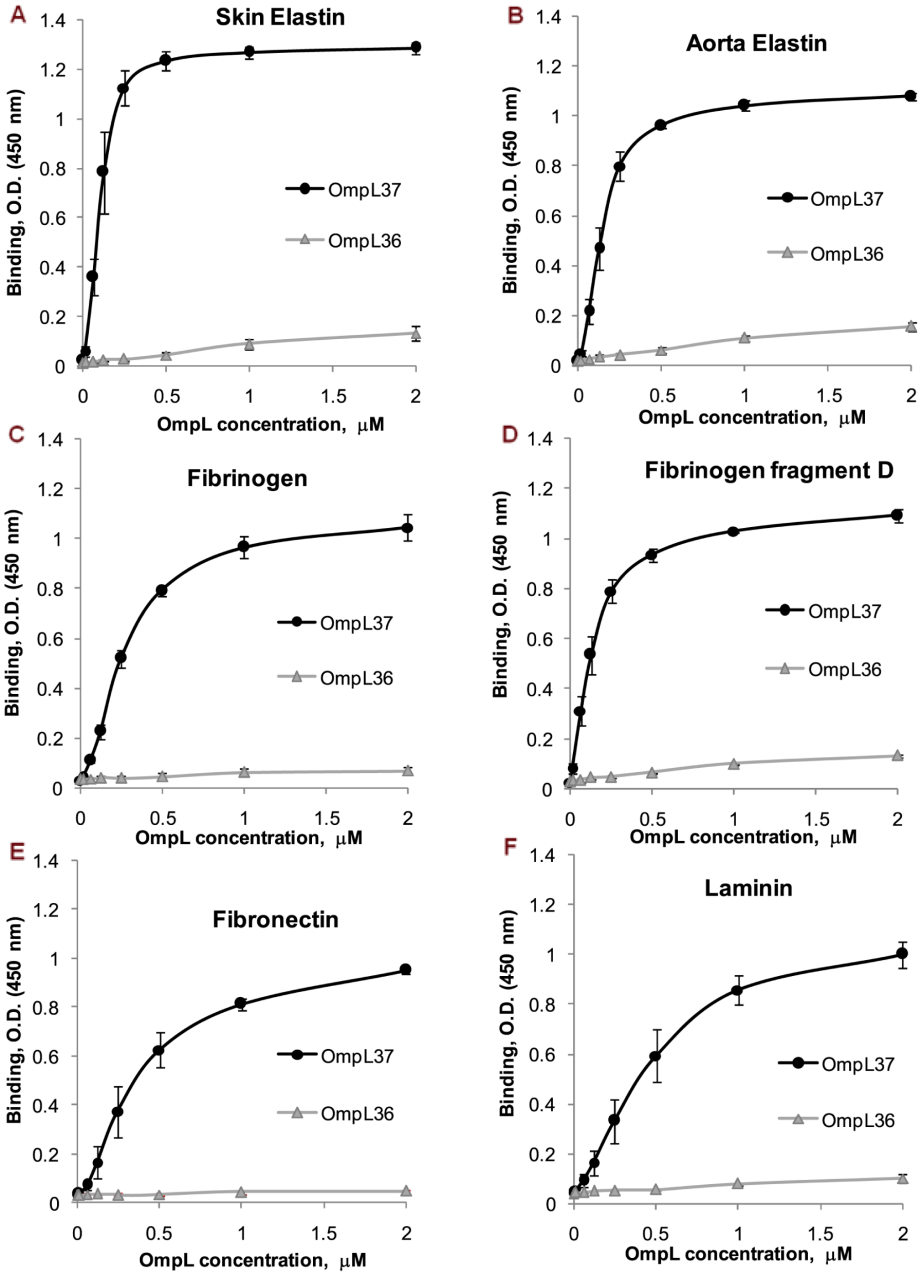
Binding to skin and aorta elastin, fibrinogen, fibrinogen fragment D, fibronectin, and laminin as a function of OmpL37 concentration. Binding of OmpL37 (concentration ranging from 0 to 2 µM) to 1 µg of immobilized (A) human skin elastin, (B) human aorta elastin, (C) human plasma fibrinogen, (D) human plasma fibrinogen fragment D, (E) human plasma fibronectin, and (F) murine laminin was measured by ELISA. The mean optical density at 450 nm ± the standard deviation of three independent experiments is shown at each point. The apparent K_d_ for saturating binding was estimated as the concentration of recombinant OmpL37 resulting in half-maximal binding (see text and Table 1). OmpL36 served as a negative control.

In order to compare binding affinities, the interaction with immobilized skin and aorta elastin, fibrinogen, fibrinogen fragment D, plasma fibronectin, plasma fibronectin 30-kDa and 45-kDa fragments, and laminin was measured as a function of OmpL37 concentration from 0 to 2 µM (Fig. 2 and Table 1). OmpL37 exhibited very strong, saturating binding to skin elastin (K_d_ , 104±19 nM), aorta elastin (K_d_ , 152±27 nM), fibrinogen (K_d_ , 244±15 nM), and fibrinogen fragment D (K_d_ , 132±30 nM) as estimated by ELISA from three independent experiments. OmpL37 binding to plasma fibronectin (K_d_ , 359±68 nM), its 30-kDa (K_d_ , 408±94 nM) and 45-kDa fragment (K_d_ , 460±70 nM), and laminin (K_d_ , 410±81 nM) were noticeably lower (Fig. 2 and Table 1). OmpL36 was used as a negative control based on our observation that OmpL36 does not bind to host ligands (Fig. 1). The comparison of apparent K_d_s for the saturation binding of host proteins by recombinant OmpL37 is summarized in Table 1.

**Table 1:** Host tissue component binding by OmpL37.

Mean K_d_ a ± SD								
Elastin, skin^b^	Elastin, aorta	Fibrinogen	Fg-D	Fn	Fn-30 kDa	Fn-45 kDa	Laminin	BSA
104±19	152±27	244±15	132±30	359±68	408±94	460±70	410±81	NS

a Estimated as nanomolar concentration at half-maximal binding by OmpL37. NS, not saturating.

b Abbreviations: Fg-D, fibrinogen fragment D; Fn, plasma fibronectin; Fn-30 kDa, plasma fibronectin 30 kDa fragment (heparin-binding domain); Fn-45 kDa, plasma fibronectin 45 kDa fragment (gelatin-binding domain).

**Figure 3:**
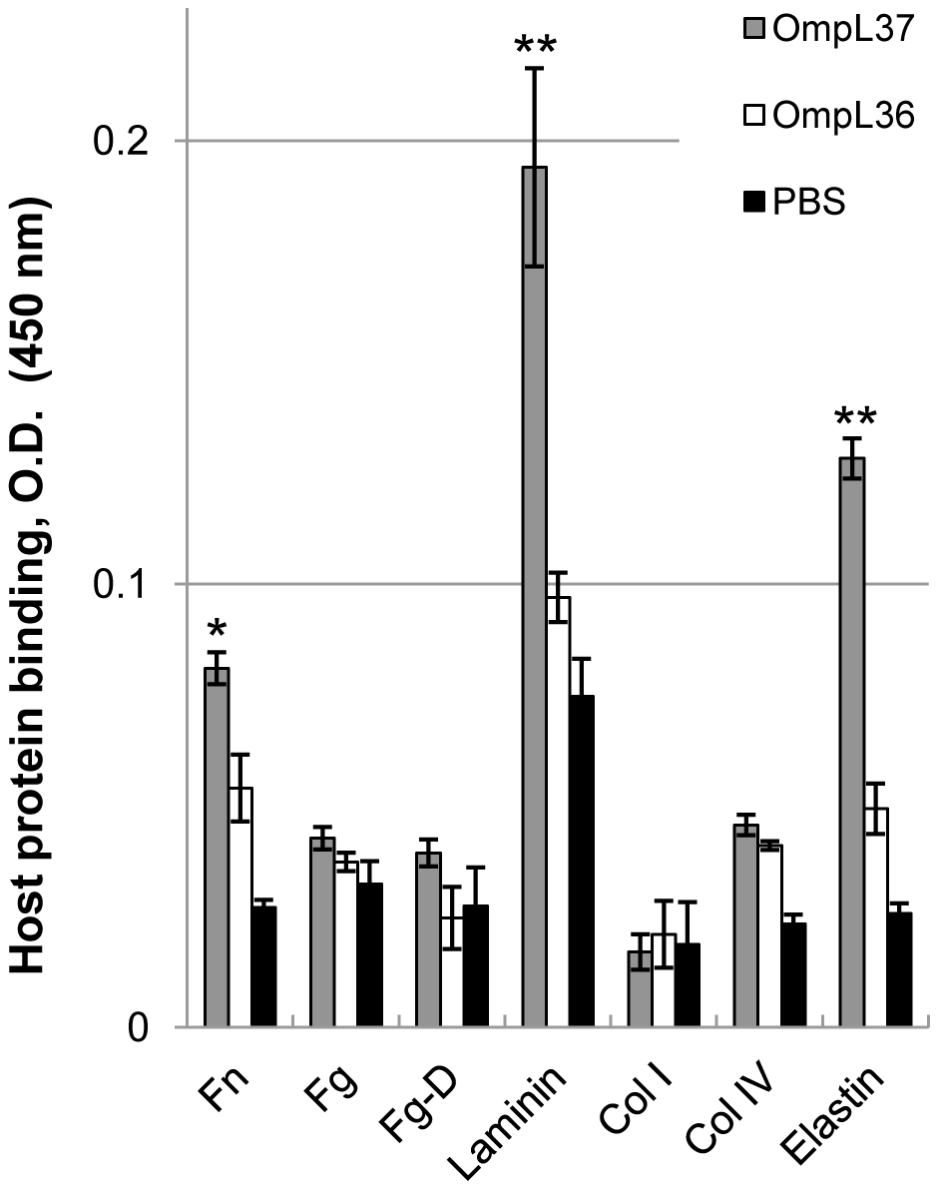
Binding of immobilized OmpL37 to freely soluble host proteins. Microtiter plates were coated with 1 µg of recombinant OmpL37 or OmpL36. One µg of plasma fibronectin (Fn), plasma fibrinogen (Fg), plasma fibrinogen fragment D (Fg-D), laminin (Lm), collagen type I (Col I), collagen type IV (Col IV), or skin elastin was added per well and binding was measured by ELISA. Data represent the mean absorbance at 450 nm ± the standard deviation from triplicate wells. The binding of recombinant OmpL37 to tissue components was compared to its binding to collagen type IV, which was chosen as a host protein control for non-specific binding based on our data in Fig. 1, by Student's two-tailed *t* test (** P<0.001, * P<0.05).

In addition, we investigated whether OmpL37 immobilized on microtiter wells bound freely soluble host proteins (Fig. 3). Immobilized OmpL37 exhibited significant binding to free laminin (P<0.001 compared to collagen IV), human skin elastin (P<0.001), and plasma fibronectin (P<0.05) (Fig. 3). It is evident that immobilized OmpL37 binds to virtually the same host proteins as freely soluble OmpL37, with the exception of fibrinogen and fibrinogen fragment D (Fig. 1 and 3). Although immobilized OmpL37 can bind to freely soluble ligands, the interaction appears to be weaker when compared to that of free OmpL37 binding to immobilized ligands (Fig. 1 and 3). This could be due to an impairment of the active conformation of OmpL37 caused by its immobilization to the microtiter wells, to the differences between antibodies utilized for detection or to the intrinsic property of less efficient binding of soluble versus immobilized host proteins, which has been described for pathogenic bacteria and their surface proteins [41]–[43].

### OmpL37 antiserum enhances leptospiral binding to skin elastin

**Figure 4:**
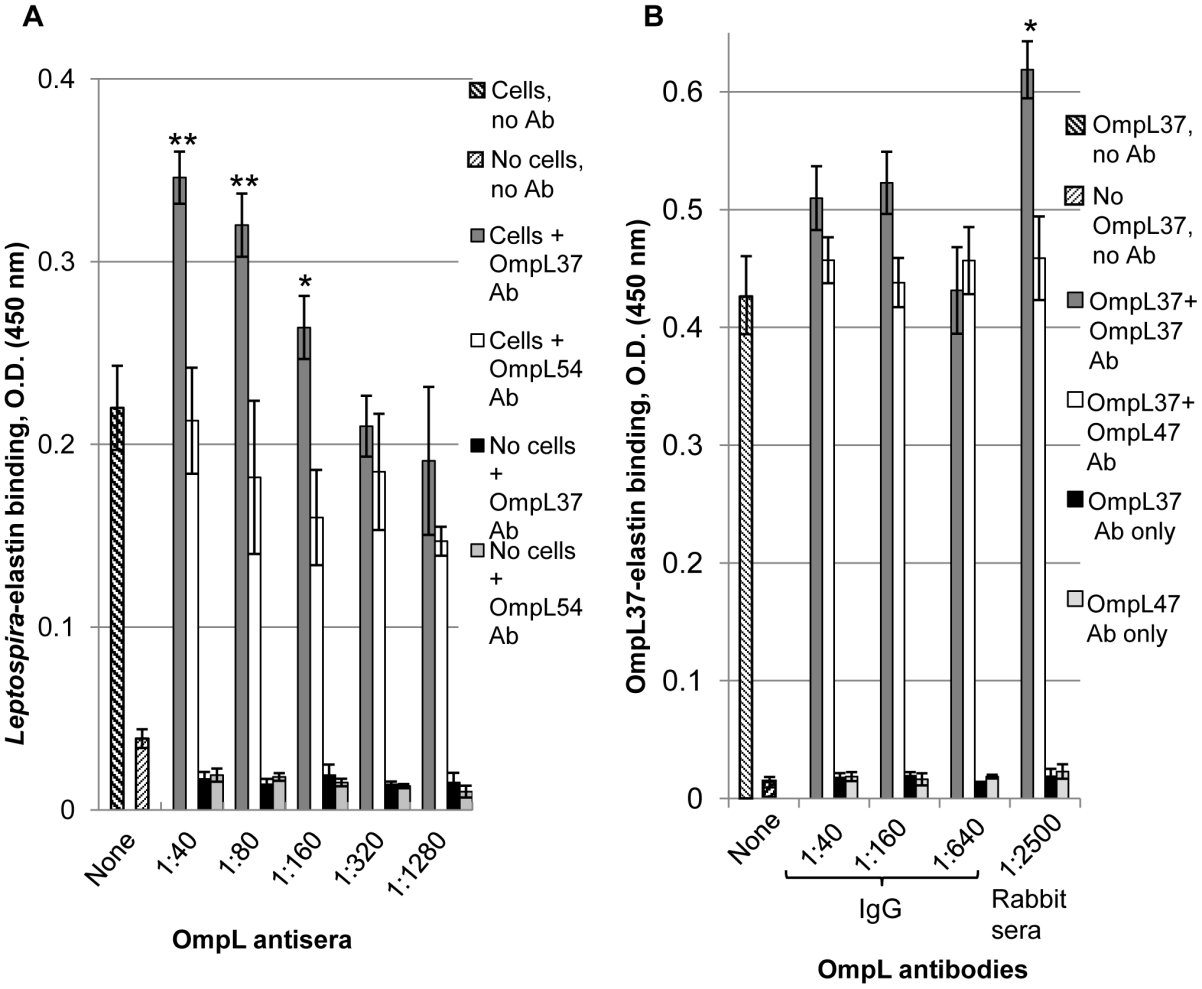
Antiserum enhancement of binding by *Leptospira* and OmpL37 to skin elastin. Microtiter wells were coated with 1 µg of human skin elastin and binding was measured by ELISA. (A) PBS-5 mM MgCl_2_ (no cells) or 1×10^8^ *L. interrogans* L1-130 in PBS-5 mM MgCl_2_ (cells) without antiserum (no Ab) or in the presence of OmpL37 or OmpL54 antiserum (Ab) diluted 1∶40, 1∶80, 1∶160, 1∶320, and 1∶1280 were added to the elastin-coated wells. (B) Recombinant OmpL37 (0.5 µg) was preincubated with or without the IgG purified from 10 times diluted OmpL37 or OmpL47 antiserum at 1∶40, 1∶160, and 1∶640 dilution and the OmpL37 or OmpL47 antiserum at 1∶2500 dilution prior to addition to elastin-coated wells. Mean absorbance at 450 nm ± the standard deviation of a representative experiment performed in triplicate is shown. Statistical significance was evaluated by one-way ANOVA comparing leptospiral or recombinant OmpL37 binding in the presence of antibodies compared with binding in the absence of antibodies (** P<0.001, * P<0.05).

We examined whether OmpL37 mediates the attachment of leptospires to human skin elastin by testing for OmpL37 antiserum inhibition of live *Leptospira* binding to immobilized skin elastin in an ELISA. Surprisingly, the OmpL37 antiserum enhanced adhesion in a dose-dependent manner, whereas antiserum for another surface-exposed OMP, OmpL54, had no effect (Fig. 4A). Leptospiral binding to skin elastin was enhanced by the OmpL37 antiserum at dilutions of 1∶40 (1.6-fold compared to no antibody, P<0.001), 1∶80 (1.5-fold, P<0.001), and 1∶160 (1.3-fold, P<0.05); the effect was not evident at higher dilutions (Fig. 4A). The increase in adhesion was not due to leptospiral agglutination as determined with a systematic examination for the presence of leptospiral aggregates or decrease in leptospiral numbers in multiple fields by dark-field microscopy (data not shown). We also investigated the effect of OmpL37 antiserum on the binding to free skin elastin by *Leptospira* immobilized on microtiter wells and found that a 1∶40 dilution of antiserum increased binding 1.4-fold (data not shown). Further, we assessed the effect of the OmpL37 antibodies on the binding of recombinant OmpL37 to skin elastin (Fig. 4B). The OmpL37 antiserum significantly enhanced recombinant OmpL37 binding to skin elastin at a dilution of 1∶2500 (1.4-fold, P<0.05), a >10-fold lower antiserum concentration compared to that required for an effect on the adhesion of leptospires (Fig. 4A and 4B). IgG purified from the OmpL37 antiserum slightly enhanced (1.2-fold) OmpL37 binding to skin elastin at dilutions of 1∶40 to 1∶160 (Fig. 4B); however the effect was not statistically significant (P>0.05), indicating the possibility that along with OmpL37-specific antibody, another serum component removed in the purification of IgG could be required for binding enhancement. The antiserum and purified IgG for another surface-exposed OMP, OmpL47, was used as a negative control and did not have any effect on OmpL37 binding (Fig. 4B). The OmpL37 antiserum did not exhibit any statistically significant effect on recombinant OmpL37 binding to fibronectin, fibrinogen, and laminin (data not shown). Further, we investigated whether convalescent leptospirosis patient sera enhance leptospiral binding to skin elastin. Dark-field microscopy revealed leptospiral agglutination by patient sera (data not shown), which prevented the assessment of leptospiral adhesion. We also investigated the effects of convalescent leptospirosis patient sera and *Leptospira*-infected hamster sera on recombinant OmpL37 binding to skin elastin but no statistically significant enhancement was observed (Fig. S1 and S2). We also tested for the inhibition of leptospiral binding to skin elastin by pre-treating the wells with a saturating concentration (0.5 µM) of recombinant OmpL37 alone or pre-incubated with the OmpL37 antiserum and found no effect (Fig. S3), indicating that additional leptospiral surface proteins could mediate elastin binding or that the avidities of recombinant and native cellular OmpL37 are significantly different.

### OmpL37 is recognized by the immune system of infected hosts

**Figure 5:**
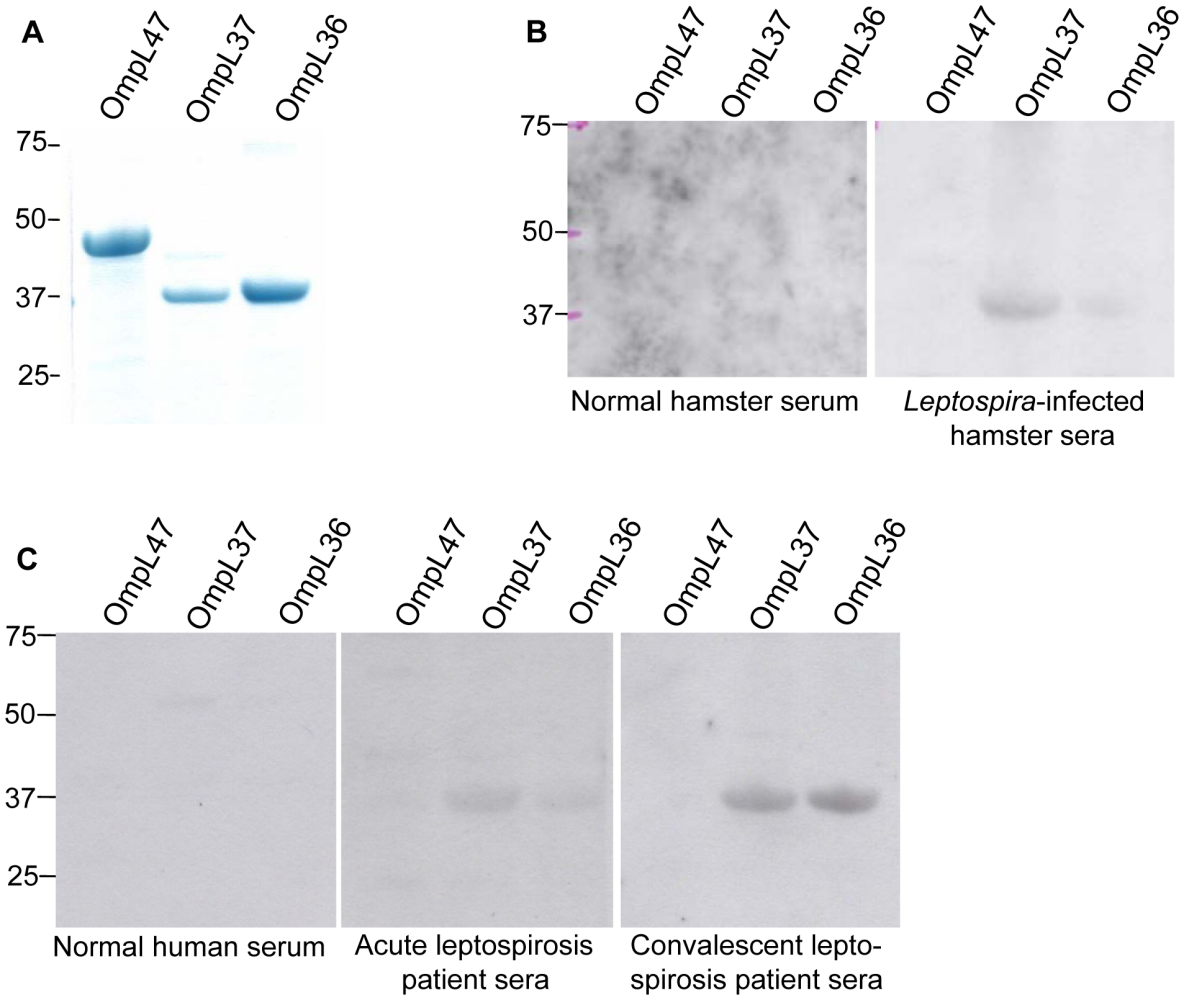
Host immune response to OmpL proteins. Recombinant OmpL47, OmpL37, and OmpL36 were separated by gel electrophoresis (2.5 µg per lane), stained with Coomassie G-250 (A) or blotted onto PVDF membrane, and analyzed for serum reactivity. (B) Membranes probed with a pool of two *L. interrogans* L1-130*-*infected hamster sera (1∶1000) or non-infected control hamster serum (1∶1000). (C) Membranes probed with normal, acute, or convalescent pooled human sera (1∶300). The positions of molecular mass standards (in kilodaltons) are indicated on the left.

To investigate whether OmpL37 can elicit an immune response from an infected host, we examined the reactivity of sera from *L. interrogans* L1-130-infected hamsters and leptospirosis patients to recombinant OmpL37 by immunoblot (Fig. 5). The results show that sera pooled from two L1-130-infected hamsters recognized OmpL37 and OmpL36 but not OmpL47 (Fig. 5B). Control hamster serum obtained after sham infection with sterile EMJH medium did not react to any of the proteins tested (Fig. 5B). Similar results were obtained when sera from acute and convalescent leptospirosis patients were tested (Fig. 5C). Pooled sera from 13 individuals diagnosed with either acute leptospirosis or recovering from the disease recognized OmpL37 and OmpL36, while no significant reactivity was found for OmpL47 and pooled normal human serum did not react to any of these proteins (Fig. 5C).

### OmpL37 is detected in pathogenic but not saprophytic *Leptospira* serovars

**Figure 6:**
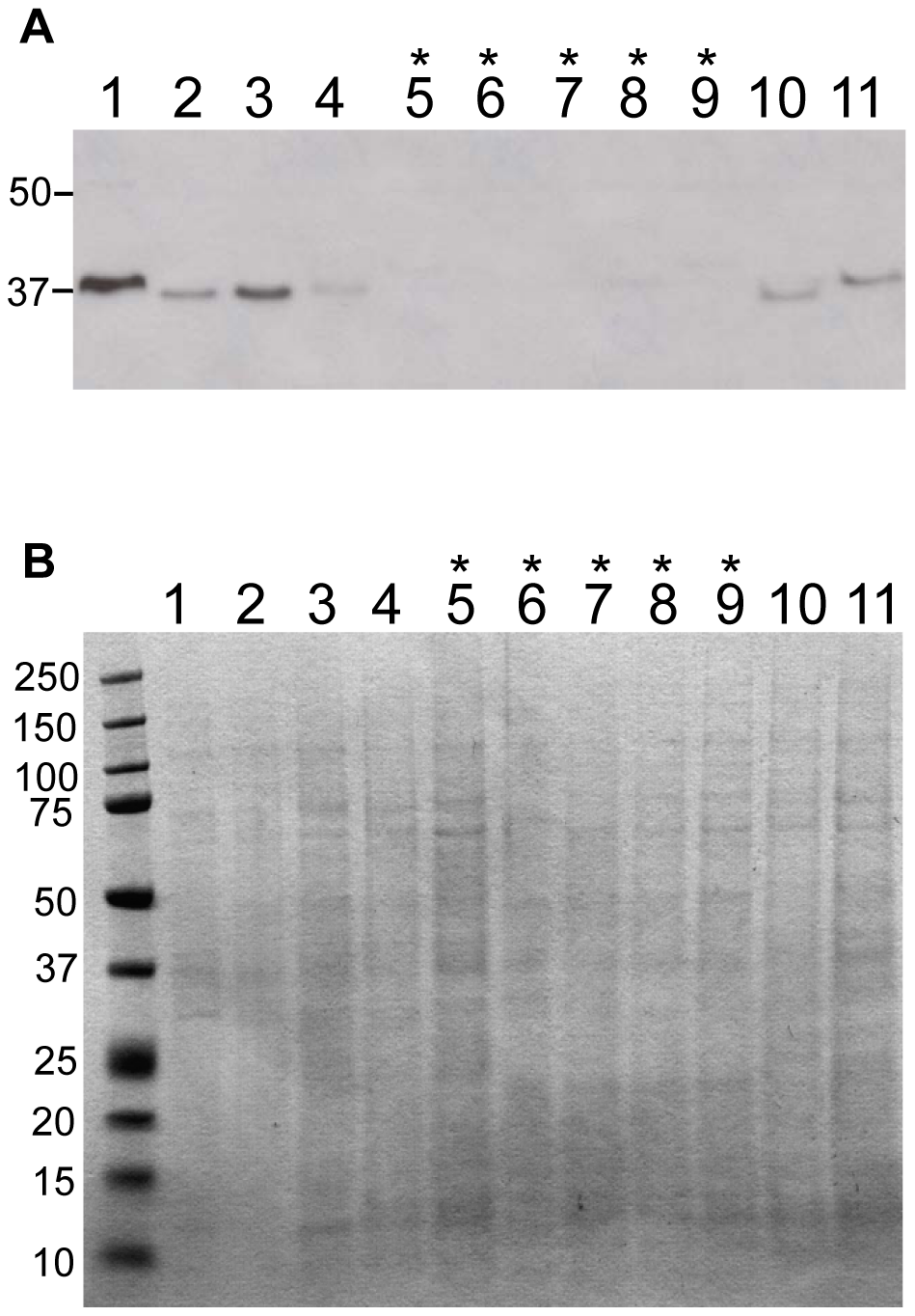
OmpL37 expression in pathogenic and saprophytic isolates of *Leptospira* serovars. Extracts of 5×10^7^ leptospires per lane were separated by gel electrophoresis, blotted onto PVDF membrane, and probed with OmpL37 antiserum (1∶1000) (A) or stained with Coomassie G-250 (B). *L. interrogans* serovar Copenhageni strain Fiocruz L1-130 (Lane 1), *L. interrogans* serovar Pomona strain PO-01 (Lane 2), *L. kirscheneri* serovar Mazdok strain 5621 (Lane 3), *L. kirscheneri* serovar Grippotyphosa strain RM 52 (Lane 4), *L. weilii* serovar Celledoni strain Celledoni (Lane 5), *L. biflexa* serovar Patoc strain Patoc 1 (Lane 6), *L. wolbachii* serovar Biflexa strain codice (Lane 7), *Leptonema illini* strain 3055 (Lane 8), *Leptospira inadai* serovar Lyme strain 10 (Lane 9), *L. borgpetersenii* serovar Tarrasovi strain Perepelicin (Lane 10), *L. borgpetersenii* serovar Javanica strain Veldrat Bataviae 46 (Lane 11). The saprophytic species in lanes 5 to 9 are indicated by asterisks. The positions of molecular mass standards (in kilodaltons) are indicated on the left.

The expression of OmpL37 by pathogenic and saprophytic *Leptospira* serovars was investigated by immunoblot using OmpL37 antiserum (Fig. 6A). Pathogenic *Leptospira* included *L. interrogans* serovars Copenhageni strain Fiocruz L1-130 and Pomona strain PO-01, *L. kirschneri* serovars Mazdok strain 5621 and Grippotyphosa strain RM 52, and *L. borgpetersenii* serovars Tarrasovi strain Perepelicin and Javanica strain Veldrat Bataviae 46. Saprophytic *Leptospira* and *Leptonema* included *Leptonema illini* strain 3055, *Leptospira weilii* serovar Celledoni strain Celledoni, *L. wolbachii* serovar Biflexa strain codice, *L. inadai* serovar Lyme strain 10, and *L. biflexa* serovar Patoc strain Patoc 1. Loading of equal amounts of proteins from whole cell lysates was confirmed by Coomassie Brilliant G-250 staining (6B). As expected, the immunoblot revealed the highest reactivity of OmpL37 antibodies against *L. interrogans* L1-130 (Fig. 6A). OmpL37 was detected in all other pathogenic *Leptospira* strains investigated with various levels of band intensity reflecting variations in either expression or antiserum reactivity (Fig. 6A). Interestingly, the OmpL37 homolog in *L. interrogans* serovar Pomona (LIP_1392, 99% identity) is expressed in considerably lower amounts (Fig. 6A). OmpL37 was not detected in any saprophytic strains investigated, with the exception of *Leptonema illini*, where a very weak band could be detected (Fig. 6A). In *L. biflexa* a homolog of OmpL37 has been annotated (LBF_0995), but the amino acid sequence reveals only 47% identity (Fig. S4), and is either not recognized by the OmpL37 antibodies or is not expressed in *L. biflexa* under the *in vitro* conditions used (Fig. 6A). The genomes of the other *Leptospira* serovars we tested have not been sequenced precluding identification of OmpL37 homologues in those organisms. Taken together, our results show that OmpL37 was detectable only in pathogenic *Leptospira* spp.

## Discussion

Bacterial adhesins are surface-exposed OMPs, often playing roles as determinants of pathogenicity that allow bacteria to colonize host tissues by attaching to host molecules, such as ECM proteins [14], [15]. *L. interrogans* is an extracellular pathogen that enters the susceptible host through skin abrasions and mucous membranes, disseminates through the bloodstream, and colonizes kidneys, lungs, and other organs. The capacity of *L. interrogans* to adhere to tissues of various organs requires surface-exposed proteins with high affinity for host cell-surface and ECM components. The abundance of elastin in the inner layers of skin (reticular region of the dermis), blood vessels, and lungs implies that *Leptospira* have the ability to recognize and attach to elastin. Whereas various leptospiral proteins have been shown to have the capacity to bind multiple ECM components, such as laminin, fibronectin, fibrinogen, and collagens [18], [19], [22]–[30], [32], only the LigB repeated domains have been shown to exhibit elastin-binding activity [21]. Thus, it is of interest to identify any additional elastin-binding proteins. We previously identified four novel surface-exposed transmembrane OMPs, OmpL36, OmpL37, OmpL47, and OmpL54 [33] and have been investigating whether these proteins bind to host proteins. The recombinant OmpL36, OmpL37, and OmpL47 proteins are soluble and suitable for functional analysis by ELISA.

The binding activity of OmpL37 for the host components tested varied widely, with the most significant binding to human skin elastin, followed by human aorta elastin, laminin, fibrinogen fragment D, fibrinogen, and plasma fibronectin (Fig. 1). The weakest binding was observed for chondroitin sulfate, heparan sulfate, and collagen type IV (Fig. 1). Although we used BSA as a widely accepted negative control for host-ligand binding, it is important to note that albumin occurs in blood and the OmpL37 binding observed with BSA and fetuin may be important in host-pathogen interactions that require further investigation. This is the first report of a leptospiral protein showing pronounced specificity for human skin elastin. The strong dose-dependent, saturating binding to skin elastin gave an estimated apparent K_d_ of 104±19 nM (Fig. 2 and Table 1), which is one of the highest affinities for all leptospiral ligand-binding proteins investigated to date, comparable to that of LigB U1 binding to fibronectin and fibrinogen with K_d_ of 72.6±11.7 nM and 87.1±2.4 nM, respectively [18], LenB binding to fibronectin and laminin with K_d_ of 106±8 nM and 118±39 nM, respectively [30], and LigB Cen binding to lung elastin with K_d_ of 101±11 nM [21]. It is apparent that LigB exhibits strongest affinity for fibronectin and fibrinogen when compared to its affinity for lung elastin and other host proteins tested [18], [21]. It is noteworthy that OmpL37 showed much stronger affinity for human skin and aorta elastin (Fig. 2 and Table 1) compared to the binding of lung elastin by multiple parts of LigB, with the exception of LigB Cen [21].

Dose-dependent, saturating binding by OmpL37 was also observed for fibrinogen and its fragment D, with considerably weaker activity for plasma fibronectin and its 30-kDa and 45-kDa fragments (Fig. 2 and Table 1). Note that whereas OmpL37 seemed to interact very efficiently with laminin (Fig. 1), the reaction kinetics revealed barely saturating binding with K_d_ greater than 410±81 nM (Fig. 2 and Table 1). Our results show that OmpL37 interacts with multiple host proteins, a property that is common with previously characterized ligand-binding proteins of *Leptospira* [18], [19], [22]–[30], [32].

*L. interrogans* was recently shown to bind to elastin *in vitro* and LigB has been attributed a partial role in this binding, and it was proposed that there are additional elastin-binding proteins [21]. This observation and our results showing strong elastin binding by recombinant OmpL37 prompted us to investigate the contribution of OmpL37 to leptospiral adherence to elastin. The addition of saturating amounts of recombinant OmpL37 did not affect *L. interrogans* binding to immobilized human skin elastin, raising the possibility that the avidity of recombinant OmpL37 for elastin is different from that of the native OmpL37 on the cell surface, affecting the ability of the recombinant protein to compete with native OmpL37. However, we observed significant increases in leptospiral elastin binding in the presence of rabbit antiserum for OmpL37, with the effect being concentration dependent (Fig. 4A). In contrast, antiserum for the surface-exposed OmpL54 did not affect elastin binding by the spirochetes (Fig. 4A), suggesting that antiserum specific for OmpL37 can promote the interaction of OmpL37 on the cell surface with elastin. Convalescent leptospirosis patient sera were also tested for effects on leptospiral binding to elastin, but the strong agglutinating effect prevented assessment of leptospiral adhesion.

Further, the OmpL37 antiserum but neither leptospirosis patient sera nor *Leptospira*-infected hamster sera enhanced the binding of recombinant OmpL37 to immobilized skin elastin (Fig. 4B and Fig. S1–S2). The discrepancy might be due to the different nature of the antibodies for OmpL37, with antibodies in rabbit serum capable of recognizing additional epitopes not recognized by antibodies generated in the infected hosts. As with the enhancement of leptospiral adhesion, the effect on recombinant OmpL37 was also specific for the OmpL37 antiserum, since rabbit antiserum for OmpL47 did not produce an effect (Fig. 4B). Interestingly, purified IgG specific for OmpL37 did not exhibit statistically significant enhancement compared to that of antiserum for OmpL37 (Fig. 4B). This discrepancy did not appear to be due to inactivation of IgG molecules as we confirmed their recognition of OmpL37 by immunoblot (data not shown). However, we cannot exclude the possibility that some level of degradation or partial loss of IgG activity could have occurred. The possibility that the enhancement effect is due to another Ig type is not likely as the IgG purification process also yields up to 20% of IgA and IgM molecules. Since antisera for other surface-exposed leptospiral proteins did not exhibit enhancement (Fig. 4A and B), we hypothesize that another serum component in addition to OmpL37 antibody might be required to enhance OmpL37 binding to elastin. Further, the OmpL37 antiserum did not enhance recombinant OmpL37 binding to fibronectin, fibrinogen, and laminin (data not shown), suggesting that this effect is specific to elastin. Finally, the OmpL37 antiserum enhanced immobilized *Leptospira* binding to freely soluble skin elastin (data not shown).

The enhancement of adhesin binding to host ligands by antibodies has been observed previously for fibronectin-binding protein FnbA of *Streptococcus dysgalactiae* [44], fibronectin-binding protein FnBPA of *Staphylococcus aureus* [45], and Lewis X (Le^x^) expressed by both human gastric mucosa and *Helicobacter pylori* [46]. Antibodies recognizing the fibronectin-binding site, Au, of FnbA bound to Au only in the presence of fibronectin [44]. Moreover, the Au antibodies enhanced ligand binding by recombinant proteins or synthetic peptides containing the Au sequence, suggesting that the antibodies can stabilize the ligand-adhesin complex, resulting in enhanced fibronectin binding. This would provide an advantage to the pathogen, where antibodies could enhance adherence to host tissue rather than inhibit this critical step in tissue colonization [44]. Interestingly, although the fibronectin-binding repeats of FnBPA are recognized weakly by antibodies in the absence of fibronectin, epitopes induced in FnBPA by its interaction with fibronectin greatly enhance its recognition by both monoclonal antibodies and sera from patients with staphylococcal infections [45]. In *H. pylori* it has been shown that Le^x^ antibodies can specifically increase bacterial adhesion [46]. This potentially positive effect on colonization could be due to an increase in bacterial aggregation or to the antibodies mediating a bivalent *H. pylori* Le^x^ – antibody– human gastric mucosa Le^x^ interaction that forms a bridge between bacteria and host cells [46]. Our interesting but somewhat surprising finding that OmpL37 antiserum enhances leptospiral binding suggests that OmpL37 contributes to the adhesion of leptospires to skin elastin. Since the OmpL37 antibodies do not promote leptospiral agglutination, we hypothesize that they in conjunction with another serum component alter the conformation of OmpL37 to promote more efficient binding to elastin. Alternatively, the antibodies may stabilize the elastin-OmpL37 complex as described for FnbA [44], resulting in enhanced elastin binding and leptospiral adhesion. The exact mechanism and functional role of this enhancement needs further investigation, which is currently under way in our laboratory.

To support our hypothesis that antibodies against OmpL37 may aid *Leptospira* during the infection process, we wanted to verify that there is a host immune response towards OmpL37. We found that an antibody response to OmpL37 occurs in both the hamster model and leptospirosis patients (acute and convalescent) (Fig. 5). Further, we investigated whether OmpL37 is expressed in pathogenic leptospiral isolates and found that OmpL37 could be detected only in pathogenic *Leptospira* serovars (Fig. 6), albeit a homologue is present in the *L. biflexa* genome (Fig. S4). Given that the amino acid sequences of OmpL37 in serovars Copenhageni and Pomona are 99% identical, the lower immunoblot reactivity indicates that OmpL37 is expressed at much lower levels in serovar Pomona (Fig. 6).

It has been reported that OmpL36 (LIC13166) and OmpL47 (LIC13050) are potential virulence factors of *L. interrogans* that are recognized by leptospirosis patient sera [47]. However, we observed the recognition of OmpL36 but not OmpL47 by the sera from infected hosts (Fig. 5). This could be due to the different techniques (ELISA versus immunoblot) employed in the studies. In addition, proteomic analysis of total protein extracts separated by 2D-gel electrophoresis identified OmpL36 and OmpL47 along with other well described leptospiral OMPs in virulent *L. interrogans* serovar Pomona cultured from infected hamsters [48]. The failure of these proteomic studies to detect OmpL37 may be explained by another mass spectrometry study focusing on cellular protein concentrations in *L. interrogans* serovar Copenhageni, which has revealed that OmpL36 is the most abundant transmembrane OMP (13^th^ highest of all cell proteins), OmpL47 is very abundant (29^th^ highest), and OmpL37 is less abundant (204^th^ highest) [49]. The lower abundance of OmpL37 in serovar Copenhageni compared to OmpL36 and OmpL47 and our data showing lower expression of OmpL37 by serovar Pomona compared to serovar Copenhageni suggest that there could be insufficient OmpL37 for detection by proteomic analysis of total proteins separated by 2D-gel electrophoresis. Although OmpL47 had very little ligand-binding activity and OmpL36 did not show significant ligand-binding activity (Fig. 1), the involvement of these proteins in virulence awaits further investigation.

Based on the results presented here, we hypothesize that OmpL37 might be involved in the pathogenesis of leptospirosis. During the initial stage of infection, the strong binding affinity of OmpL37 for human skin elastin would facilitate the attachment of leptospires to an inner elastin-rich layer of the skin exposed by abrasion. Later during dissemination, the ability of OmpL37 to efficiently bind human aorta elastin suggests that OmpL37 could promote leptospiral attachment to elastin-rich vascular structures, including the walls of the pulmonary, cardiac, and other blood vessels, possibly explaining the propensity of leptospirosis to result in hemorrhagic complications [1], [50], [51]. The enhancement of leptospiral binding to elastin by OmpL37 antiserum supports the hypothesis that the host immune response to OmpL37 could promote rather than inhibit the adherence of infecting spirochetes to elastin-rich tissues, possibly also aiding in evading immunological clearance. Future studies are planned to map the elastin-binding sites in OmpL37 to further understand the antiserum enhancement effect. We also plan to identify the serum component that is apparently required in addition to OmpL37 antibodies in the enhancement of elastin-binding. Finally, we plan to study the contribution of OmpL37 during the initial and subsequent stages of leptospirosis.

## Supporting Information

Figure S1 Effects of convalescent leptospirosis patient sera on recombinant OmpL37 binding to skin elastin. Microtiter wells were coated with 1 µg of human skin elastin and binding was measured by ELISA. Recombinant OmpL37 (0.5 µg) was preincubated for 1 h at room temperature with convalescent leptospirosis patient sera or normal human sera at 1∶640, 1∶2560, and 1∶10,240 dilution prior addition to elastin-coated wells. Mean absorbance at 450 nm ± the standard deviation of a representative experiment performed in triplicate is shown.
(0.20 MB PPT)

Figure S2 Effects of *Leptospira*-infected hamster sera on recombinant OmpL37 binding to skin elastin. Microtiter wells were coated with 1 µg of human skin elastin and binding was measured by ELISA. Recombinant OmpL37 (0.5 µg) was preincubated for 1 h at room temperature with *Leptospira*-infected hamster sera or normal hamster sera at 1∶640 and 1∶2560 dilution prior addition to elastin-coated wells. Mean absorbance at 450 nm ± the standard deviation of a representative experiment performed in triplicate is shown.
(0.19 MB PPT)

Figure S3 Leptospiral binding to elastin after recombinant OmpL37 has been pre-bound to elastin in presence of OmpL37 antiserum. Microtiter wells were coated with 1 µg of human skin elastin and binding was measured by ELISA. Prior the addition of leptospires (1.4×108 per well), 0.5 µM of recombinant OmpL37 was either added directly to elastin-coated microtiter wells or pre-incubated with anti-OmpL37 or anti-OmpL47 (negative control) at a 1∶500 dilution and added to the microtiter wells. Mean absorbance at 450 nm ± the standard deviation of a representative experiment performed in triplicate is shown. Statistical significance was evaluated by one-way ANOVA comparing leptospiral binding without recombinant OmpL37 compared with binding after addition of recombinant OmpL37 (P>0.05).
(0.17 MB PPT)

Figure S4 Amino acid sequence homology of *L. interrogans* OmpL37 (LIC12263) and *L. biflexa* LBF 0995. BLAST analysis was performed between *L. interrogans* LIC12263 (OmpL37) and *L. biflexa* genome. The highest scored homolog, LBF 0995 had 154/326 identities (47%), 219/326 positives (67%) and 14/326 gaps (4%).
(0.11 MB PPT)
