# Supplementary figures and images for: The OmpL37 Surface-Exposed Protein Is Expressed by Pathogenic Leptospira during Infection and Binds Skin and Vascular Elastin
Source: PLoS Negl Trop Dis. 2010 Sep 7;4(9):e815. doi: 10.1371/journal.pntd.0000815 (PMC2935396; doi:10.1371/journal.pntd.0000815)

## Slide 1
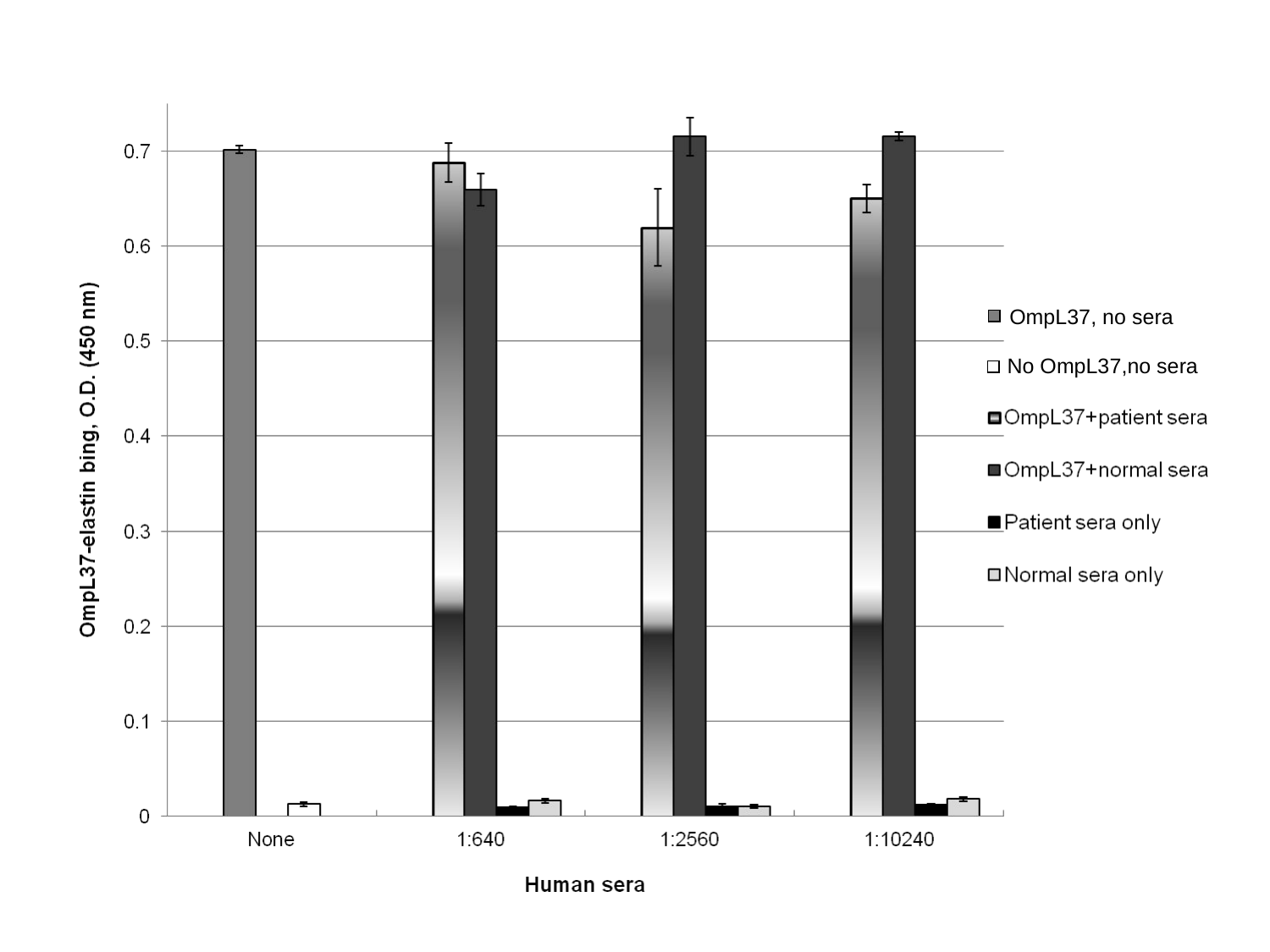

OmpL37, no sera
No OmpL37,no sera

Supplement: Figure S1 — Effects of convalescent leptospirosis patient sera on recombinant OmpL37 binding to skin elastin. Microtiter wells were coated with 1 µg of human skin elastin and binding was measured by ELISA. Recombinant OmpL37 (0.5 µg) was preincubated for 1 h at room temperature with convalescent leptospirosis patient sera or normal human sera at 1∶640, 1∶2560, and 1∶10,240 dilution prior addition to elastin-coated wells. Mean absorbance at 450 nm ± the standard deviation of a representative experiment performed in triplicate is shown. (0.20 MB PPT) [file pntd.0000815.s001.ppt]

## Slide 1
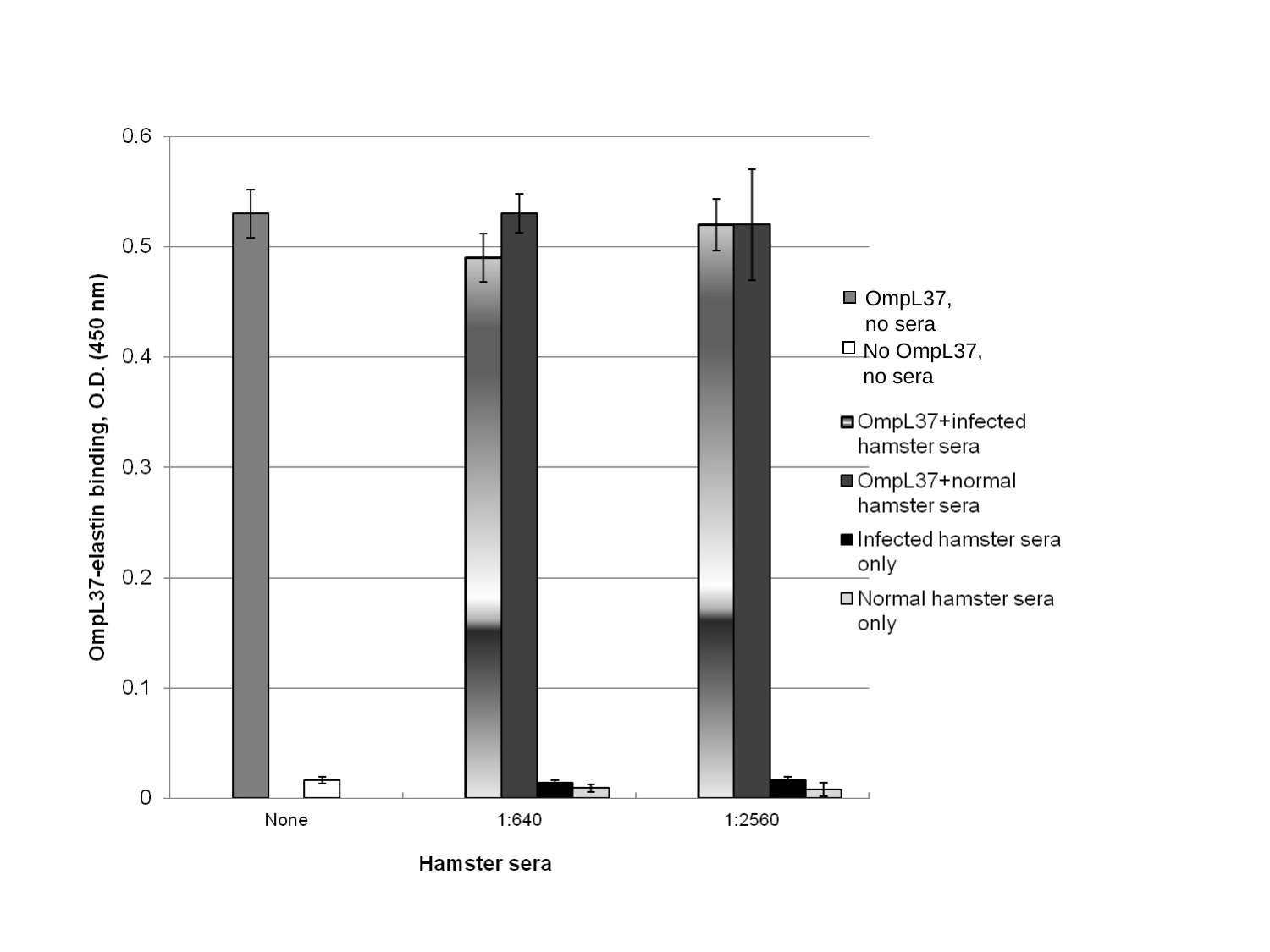

OmpL37,
no sera
No OmpL37,
no sera

Supplement: Figure S2 — Effects of Leptospira-infected hamster sera on recombinant OmpL37 binding to skin elastin. Microtiter wells were coated with 1 µg of human skin elastin and binding was measured by ELISA. Recombinant OmpL37 (0.5 µg) was preincubated for 1 h at room temperature with Leptospira-infected hamster sera or normal hamster sera at 1∶640 and 1∶2560 dilution prior addition to elastin-coated wells. Mean absorbance at 450 nm ± the standard deviation of a representative experiment performed in triplicate is shown. (0.19 MB PPT) [file pntd.0000815.s002.ppt]

## Slide 1
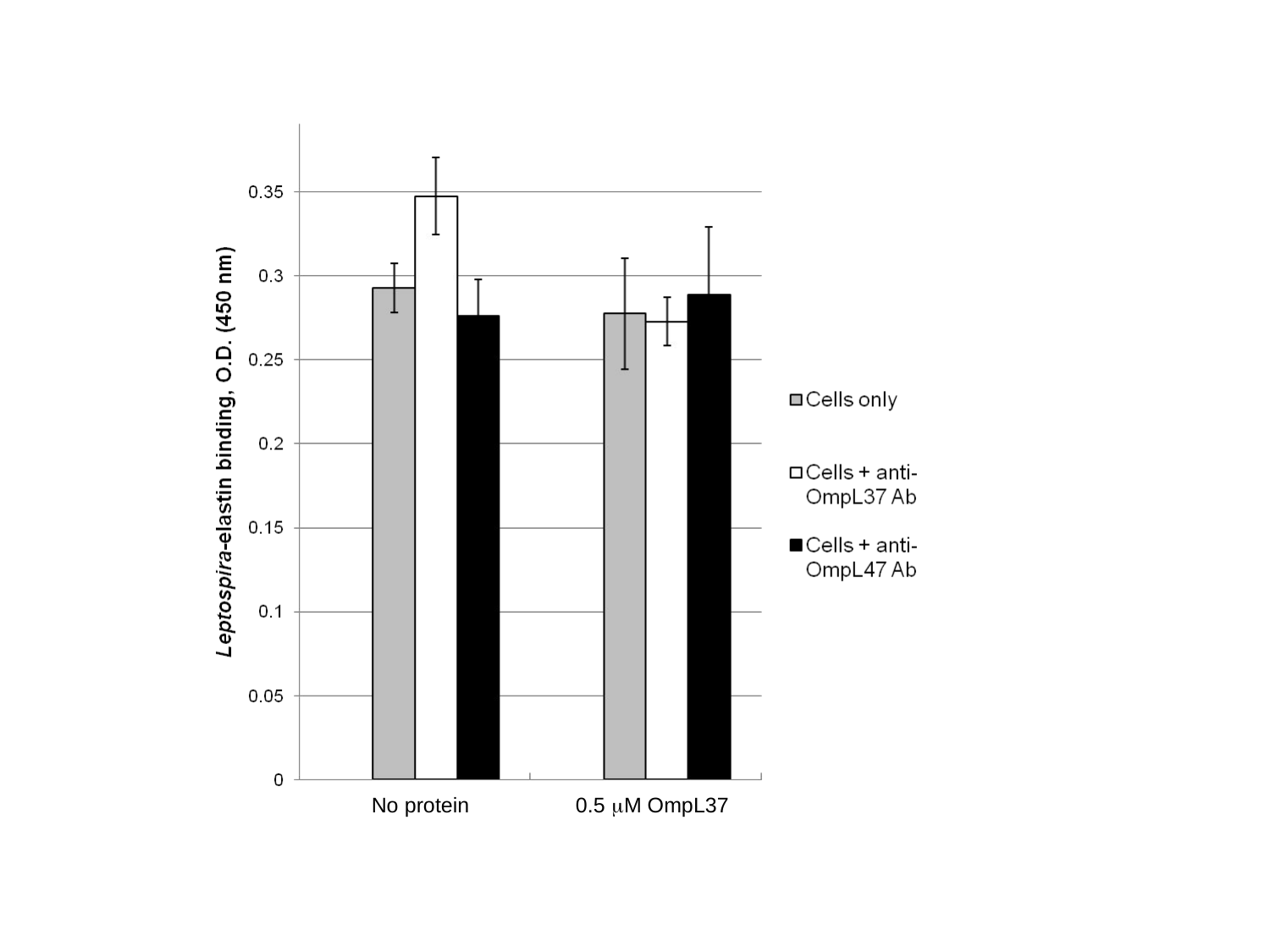

No protein 0.5 μM OmpL37

Supplement: Figure S3 — Leptospiral binding to elastin after recombinant OmpL37 has been pre-bound to elastin in presence of OmpL37 antiserum. Microtiter wells were coated with 1 µg of human skin elastin and binding was measured by ELISA. Prior the addition of leptospires (1.4×10^8^ per well), 0.5 µM of recombinant OmpL37 was either added directly to elastin-coated microtiter wells or pre-incubated with anti-OmpL37 or anti-OmpL47 (negative control) at a 1∶500 dilution and added to the microtiter wells. Mean absorbance at 450 nm ± the standard deviation of a representative experiment performed in triplicate is shown. Statistical significance was evaluated by one-way ANOVA comparing leptospiral binding without recombinant OmpL37 compared with binding after addition of recombinant OmpL37 (P>0.05). (0.17 MB PPT) [file pntd.0000815.s003.ppt]
