# Supplementary material for: The OmpL37 Surface-Exposed Protein Is Expressed by Pathogenic Leptospira during Infection and Binds Skin and Vascular Elastin
Source: PLoS Negl Trop Dis. 2010 Sep 7;4(9):e815. doi: 10.1371/journal.pntd.0000815 (PMC2935396; doi:10.1371/journal.pntd.0000815)
Supplement: Figure S4 — Amino acid sequence homology of L. interrogans OmpL37 (LIC12263) and L. biflexa LBF 0995. BLAST analysis was performed between L. interrogans LIC12263 (OmpL37) and L. biflexa genome. The highest scored homolog, LBF 0995 had 154/326 identities (47%), 219/326 positives (67%) and 14/326 gaps (4%). (0.11 MB PPT) [file pntd.0000815.s004.ppt]

## Slide 1
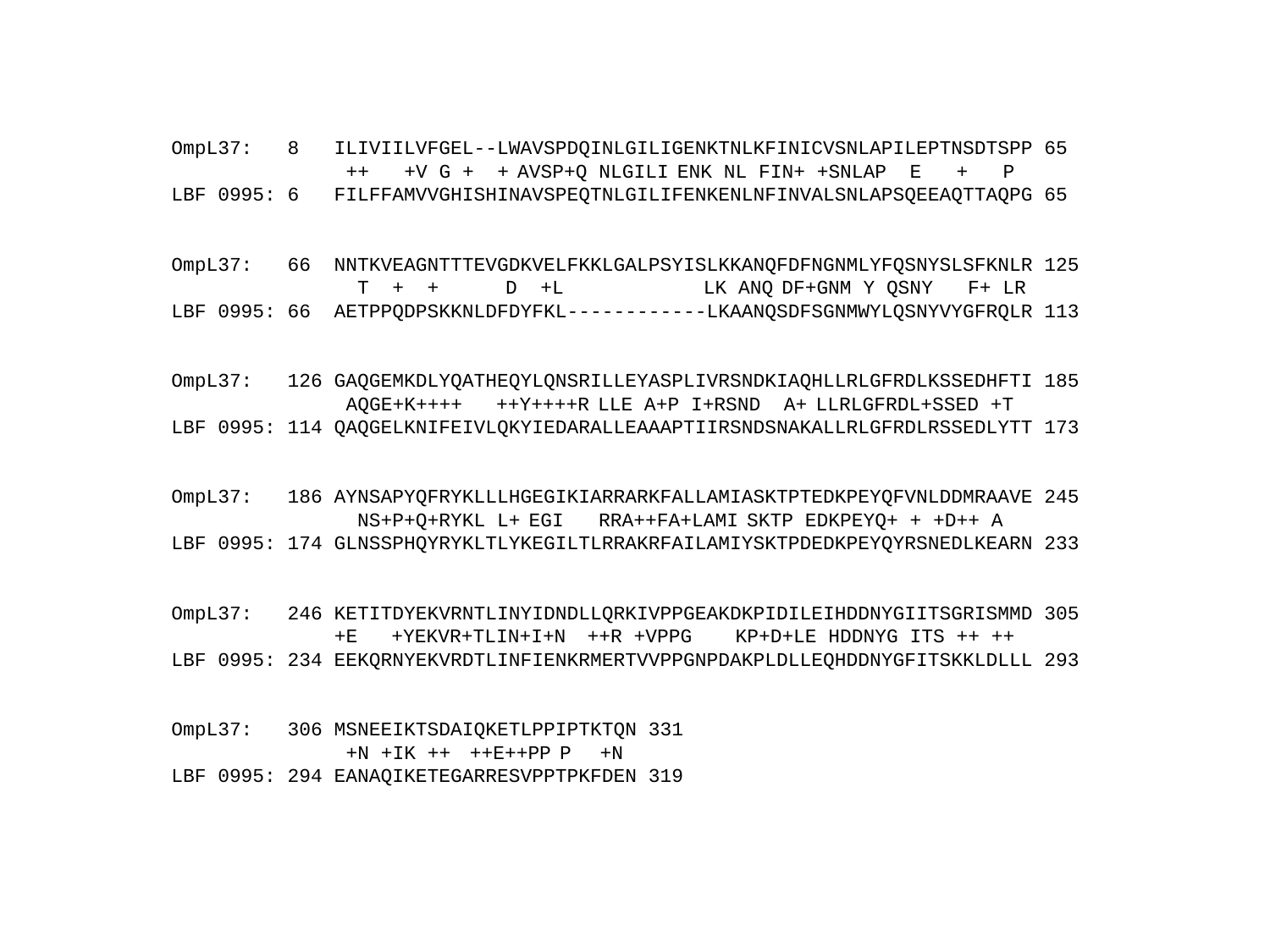

OmpL37: 8 ILIVIILVFGEL--LWAVSPDQINLGILIGENKTNLKFINICVSNLAPILEPTNSDTSPP 65
 ++ +V G + + AVSP+Q NLGILI ENK NL FIN+ +SNLAP E + P
LBF 0995: 6 FILFFAMVVGHISHINAVSPEQTNLGILIFENKENLNFINVALSNLAPSQEEAQTTAQPG 65
OmpL37: 66 NNTKVEAGNTTTEVGDKVELFKKLGALPSYISLKKANQFDFNGNMLYFQSNYSLSFKNLR 125
 T + + D +L LK ANQ DF+GNM Y QSNY F+ LR
LBF 0995: 66 AETPPQDPSKKNLDFDYFKL------------LKAANQSDFSGNMWYLQSNYVYGFRQLR 113
OmpL37: 126 GAQGEMKDLYQATHEQYLQNSRILLEYASPLIVRSNDKIAQHLLRLGFRDLKSSEDHFTI 185
 AQGE+K++++ ++Y++++R LLE A+P I+RSND A+ LLRLGFRDL+SSED +T
LBF 0995: 114 QAQGELKNIFEIVLQKYIEDARALLEAAAPTIIRSNDSNAKALLRLGFRDLRSSEDLYTT 173
OmpL37: 186 AYNSAPYQFRYKLLLHGEGIKIARRARKFALLAMIASKTPTEDKPEYQFVNLDDMRAAVE 245
 NS+P+Q+RYKL L+ EGI RRA++FA+LAMI SKTP EDKPEYQ+ + +D++ A
LBF 0995: 174 GLNSSPHQYRYKLTLYKEGILTLRRAKRFAILAMIYSKTPDEDKPEYQYRSNEDLKEARN 233
OmpL37: 246 KETITDYEKVRNTLINYIDNDLLQRKIVPPGEAKDKPIDILEIHDDNYGIITSGRISMMD 305
 +E +YEKVR+TLIN+I+N ++R +VPPG KP+D+LE HDDNYG ITS ++ ++
LBF 0995: 234 EEKQRNYEKVRDTLINFIENKRMERTVVPPGNPDAKPLDLLEQHDDNYGFITSKKLDLLL 293
OmpL37: 306 MSNEEIKTSDAIQKETLPPIPTKTQN 331
 +N +IK ++ ++E++PP P +N
LBF 0995: 294 EANAQIKETEGARRESVPPTPKFDEN 319
